# Golden Standard or Obsolete Method? Review of ECG Applications in Clinical and Experimental Context

**DOI:** 10.3389/fphys.2022.867033

**Published:** 2022-04-25

**Authors:** Tibor Stracina, Marina Ronzhina, Richard Redina, Marie Novakova

**Affiliations:** ^1^ Department of Physiology, Faculty of Medicine, Masaryk University, Brno, Czech Republic; ^2^ Department of Biomedical Engineering, Faculty of Electrical Engineering and Communication, Brno University of Technology, Brno, Czech Republic; ^3^ International Clinical Research Center, St. Anne’s University Hospital Brno, Brno, Czech Republic

**Keywords:** electrocardiogram, ECG recording, animal model, deep learning, ECG analysis, artificial intelligence, isolated heart, arrhythmia classification

## Abstract

Cardiovascular system and its functions under both physiological and pathophysiological conditions have been studied for centuries. One of the most important steps in the cardiovascular research was the possibility to record cardiac electrical activity. Since then, numerous modifications and improvements have been introduced; however, an electrocardiogram still represents a golden standard in this field. This paper overviews possibilities of ECG recordings in research and clinical practice, deals with advantages and disadvantages of various approaches, and summarizes possibilities of advanced data analysis. Special emphasis is given to state-of-the-art deep learning techniques intensely expanded in a wide range of clinical applications and offering promising prospects in experimental branches. Since, according to the World Health Organization, cardiovascular diseases are the main cause of death worldwide, studying electrical activity of the heart is still of high importance for both experimental and clinical cardiology.

## 1 Introduction

Cardiovascular disorders are the major cause of death in developed countries. Due to the change of lifestyle, their incidence increases recently also in countries where cardiovascular morbidity and consequent mortality have not been considered a problem until now. Enormous economic and social burden of such situation promotes further research of both physiological and pathophysiological cardiovascular processes.

Cardiac action starts with electrical event—membrane depolarization—which is then followed by a mechanical response, e.g., cardiac muscle contraction. After this event, called systole, the cardiac cycle continues with diastole (membrane repolarization and consequent muscle relaxation). Obtaining quite detailed information about electrical activity of the heart seems to be technically easier than obtaining comparably detailed information about mechanical events.

The first attempts to study electrical processes related to cardiac action were performed already in the 19th century (AlGhatrif and Lindsay, 2012), based on the experience with recording electrical current from skeletal muscles even a century earlier. The first successful recording of electrical activity of human heart was achieved in 1887 by the British physician and physiologist Augustus Waller (Besterman and Creese, 1979). His contribution to this area is so important that it is rather unfair that his name is not mentioned together with the so-called father of electrocardiography, Dutch physiologist and physician Willem Einthoven. Einthoven, a Nobel Prize winner in 1924, is highly recognized since he standardized the whole method: he introduced the term “electrocardiogram” (ECG), reported a typical ECG of a healthy man with five deflections labeled P, Q, R, S, and T, and, last but not least, built a sort of the first ECG recorder based on a string galvanometer. Soon after the first clinically relevant attempts to record the human ECG curve, the method was used by other researchers who improved the recording possibilities and thus opened a new medical field—cardiology. Worth mentioning are especially Wilson and Goldberger, who introduced the central terminal thus enabled to record unipolar leads and their augmented version. All other research in heart electrophysiology proceeded from these key steps.

Concurrently to above-described milestones of ECG development and its establishment into routine clinical use, researchers were interested in electrical changes accompanying the heart activity other than whole-body models. The fundamental approach in this area is the isolated heart model. First, successful isolation and perfusion of frog heart were achieved by Elias Cyon and Carl Ludwig (Zimmer, 1998). Then, a model of isolated perfused mammalian heart was introduced by H. Newell Martin and Oscar Langendorff. The latter invented and published in 1895 a perfusion setup which is suitable for all hearts with a coronary system. Nutrition is supplied to the heart muscle by perfusion solution, administered *via* a cannula inserted into the aorta of the fully explanted heart. The hydrostatic pressure of the solution closes the aortic valve, and solution enters the coronary arteries, leaves via sinus coronarius, and drips off.

A model of isolated heart perfused according to Langendorff (also known as Langendorff’s heart) is the first experimental setting which enables recording of electrical activity of the heart in the style which resembles the ECG (Olejnickova, Novakova, and Provaznik, 2015). Of course, there were some attempts to record electrical activity of the heart before introduction of this model; however, the quality of recording and consequently the scientific impact of such data were low.

## 2 ECG Recording

### 2.1 Clinical Perspective

From Einthoven’s first ECG recording system till now, ECG recording has undergone huge transformation from a full-analog system to fully computerized ECG recorders (Rautaharju, 2016). Regardless of technological progress, the basic clinical approach—12-lead ECG recording—is still based on Einthoven, Wilson, and Goldberg’s inventions. A standard 12-lead configuration contains three bipolar limb leads originally introduced by Einthoven (I, II, and III), three Goldberg’s augmented unipolar limb leads (aVL, aVR, aVF), and six unipolar chest leads (V1–V6), where a Wilson central terminal serves as the reference electrode. Ten skin electrodes are placed on the left wrist or arm (LA), on the right wrist or arm (RA), on the left leg (LL), on the right leg (RL), and on the chest (V1–V6). Chest leads (V1–V6) cover small part of the chest circumference. Therefore, for specific purposes, modified chest leads, shifted to different intercostal spaces or to completely different positions, are used. For example, the leads V3R–V6R are placed on the right side of the chest mirroring leads V3–V6. Such modification is useful for diagnostics of ECG abnormalities originated in the right ventricle.

The 12-lead ECG configuration gives spatial information about the cardiac electric activity (Kalra, Lowe, and Al-Jumaily, 2019). Since ECG leads have both positive and negative poles, they may be viewed from two spatial directions. The standard 12-lead ECG system presents the “positive” ECG leads in a single well-ordered sequence in the transverse plane (chest leads V1–V6), but in two separate non-anatomical sequences in the frontal plane (I–III and aVR–aVF). Moreover, the aVR lead shows the cardiac electric activity from the right-side view, but the other eleven leads show the heart activity from the left-side view. The direction of the deflections in the aVR lead tends to be opposite to that in all other leads. In such presentation, it is usually hard to integrate consideration of lead aVR into overall ECG interpretation. To cope with such problems, an orderly presented ECG system was introduced. The so-called Cabrera system is based on two modifications of the standard 12-lead ECG: 1) changing aVR polarity (–aVR) and 2) changing re-organization of limb leads in the anatomical order (aVL, I, –aVR, II, aVF, III) (Lam, Wagner, and Pahlm, 2015). Even such modification may help to easily interpret ECG changes, and only Sweden has adopted the Cabrera system as a national standard.

Reconstruction of the cardiac vector movement from the 12-lead ECG is—despite all modifications—arduous and imagination-demanding. For such analysis, orthogonal leads are much more useful. Orthogonal leads record the electric activity from three perpendicular axes of the body (horizontal, vertical, and sagittal). Frank leads are one of the most clinically relevant orthogonal systems.

The standard 12-lead system is mostly used for ambulatory ECG recording. Also, such recording is used during the exercise stress test. Ambulatory 24 h ECG monitoring—Holter monitor—is employed in diagnostics of paroxysmal cardiac events (such as paroxysmal arrhythmias). The Holter monitor may use a 12-lead system; however, modern devices record two or three modified leads only (Kalra, Lowe, and Al-Jumaily, 2019). In case of rare symptoms, an implantable loop recorder is valuable (Giada et al., 2007). Such long-term monitor is placed under the skin on the chest and can automatically record long continuous signals (up to 3 years) (Kalra, Lowe, and Al-Jumaily, 2019). Recently, several innovative methods for ECG monitoring were introduced, including patch sensors, EPIC (electric potential integrated circuit) sensors, chest harnesses, and vest shirts (Kalra, Lowe, and Al-Jumaily, 2019; Soroudi et al., 2019). A general trend is to minimize electrodes and make devices wearable, remote-controlled, and programmable.

A typical ECG curve shows five deflections labeled P, Q, R, S, and T. The P wave reflects depolarization of the atria. Deflections Q, R, and S create a thin complex—QRS complex, which represents ventricular depolarization. The R peak is always positive (pointing upward). Deflection Q is always the negative deflection preceding R peak. Deflection S is always the negative deflection following R peak. Depending on the orientation of ECG lead, some of the deflections of the QRS complex may not be expressed. The fifth deflection—the T wave—reflects repolarization of the ventricles. Between the end of P wave and the onset of QRS complex, a period of isoelectric line called the PQ segment is found. The PQ segment represents atrioventricular (AV) conduction or AV delay. The PQ segment together with the P wave forms a PQ interval. The PQ interval duration reflects propagation of depolarization from the atria to the ventricles. Between the end of QRS complex and the onset of T wave, there is a period of isoelectric line called the ST segment. During the ST segment, all the ventricular cardiomyocytes are fully depolarized, resting in the plateau phase of the action potential. Complete electric revolution of the ventricle is represented by a QT interval—the period from the onset of the QRS complex to the end of the T wave. The distance of two consecutive R peaks—RR interval—represents the duration of one electric cycle of the heart; therefore, it defines the heart rate. The amplitude of deflections as well as polarity of P and T waves depends on the orientation of ECG lead. This topic is out of the scope of this article, and therefore, it is not discussed here.

ECG recording plays an irreplaceable role in diagnostics of various cardiovascular diseases. Diagnostics of arrhythmias is completely based on an ECG record. Moreover, the QT interval prolongation—an independent risk factor for ventricular arrhythmias—can be detected exclusively from an ECG. Typical ECG changes, such as the ST segment elevation and T wave alteration, are related to coronary artery disease and myocardial infarction. According to the specific changes in the 12-lead ECG record, an occlusion of coronary artery can be localized. Also, left ventricular hypertrophy can be diagnosed with high sensitivity and specificity from the ECG record (Yu et al., 2021). Also, other cardiovascular diseases can manifest themselves as ECG curve alterations—for instance, myocarditis, pericarditis, myocardial fibrosis, amyloidosis, and inherited and acquired defects (such as dextrocardia, mitral stenosis, or regurgitation). ECG-based diagnostics of pulmonary embolism represents a specific issue. Although advanced techniques such as echocardiography, computed tomography, or magnetic resonance are widely used, the diagnostic role of ECG—especially in acute pulmonary embolism—is still highly valued by clinicians (Van Mieghem et al., 2004). However, it is necessary to emphasize that ECG signs of pulmonary embolism may be imitated by severe pneumonia or pneumothorax.

ECG may also help in diagnostics of numerous non-cardiovascular diseases (Van Mieghem et al., 2004). Typical ECG curve alterations are detectable in electrolyte imbalances, such as hyperkalemia and hypocalcemia. Both hyperthyroidism and hypothyroidism are presented by heart rate alterations and non-specific ST-T changes. Specific ECG changes are associated with various disorders of the central nervous system, e.g., subarachnoid hemorrhage, head trauma, and acute meningitis. Hypothermia and hyperthermia cause characteristic ECG alterations. Last but not least, there are many drugs with cardiovascular side effects. Drug-induced ECG alterations have to be considered during treatment by antiarrhythmics, beta-blockers, antibiotics, antihistamines, antipsychotics, and others.

Innovative approaches of ECG recording bring new challenges in ECG interpretation. Reduction of the employed electrodes leads to the reduction of ECG lead number. Recently, diagnostic potential of reduced-lead ECG was discussed during the PhysioNet/Computing in Cardiology Challenge (Reyna et al., 2021). The results suggest that two leads can be enough for some of the used diagnostics. However, the accuracy of the diagnostics depends also on the particular combination of leads (Reyna et al., 2021). On the contrary, a device which enables to record a high number of ECG leads has been introduced and successfully tested for detection of life-threatening events in a human phantom equipped with an ECG simulator (Wojcik et al., 2021).

### 2.2 Animal Perspective

The quality (sampling rate and resolution) of the ECG record is important particularly in animals of high heart rate (mice, rats).

**FIGURE 1:**
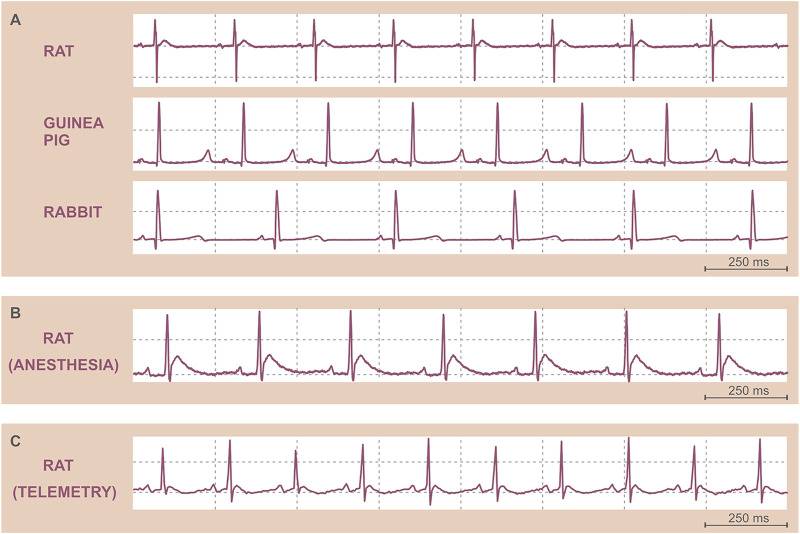
Original ECG records. **(A)** Isolated heart electrograms of various species. **(B)** ECG in an anesthetized Sprague Dawley male rat recorded by needle electrodes. **(C)** ECG in a freely moving Wistar male rat recorded by telemetry. Note the differences in the heart rate among species **(A)** and between anesthetized **(B)** and conscious **(C)** rats.

If the electric activity is recorded directly from the surface of the heart, the term “electrogram” (EG) should be preferred. In isolated heart models, originally the electric activity was recorded by needle or hook electrodes attached directly into the cardiac muscle. However, mechanical irritation caused by electrode attachment may induce ventricular arrhythmias. To minimize it, only one pair of electrodes is usually attached, and thus, one EG lead is recorded. A less-invasive approach is possible if the isolated heart is immersed into the bath containing saline (e.g., Krebs–Henseleit or Tyrode solution). Then, an EG is recorded contact-less by electrodes placed on the inner surface of the bath (Figure 1A). Various numbers and positions of electrodes may be used including a two-dimensional lead system (Ronzhina et al., 2017) or three-dimensional orthogonal lead system (Janousek et al., 2010). Such approach minimizes mechanical irritation of the heart and allows to record more than one EG lead. However, moving artifacts may compromise EG analysis.

To study more complex responses of the cardiovascular system including neuro-humoral regulatory mechanisms, a whole-body animal model has to be engaged. Various animal species are used to study heart electric activity—including zebrafish, small rodents (mouse, rat, guinea pig), rabbit, dog, pig, and others. A broad range of methods are used to record the ECG in animals. Generally, the methods can be divided into two groups: 1) methods used in anesthetized animals and 2) methods enabling ECG recording in conscious animals.

In small animals, including rodents and rabbit, the ECG is frequently recorded in general anesthesia (Ha, Oh, and Kang, 2020). In anesthetized animals, needle electrodes are usually placed under the skin of thorax or limbs. Alternatively, clip or strip electrodes can be fixed to the paws. Other non-invasive approaches, such as standard skin electrodes, are less effective due to thick fur of the animals. If non-invasive attachment of the electrodes is preferred, the fur has to be shaved. Also, contact conductive ECG gel may improve the signal transduction. The position of the electrodes usually copies standard bipolar limb leads well known in human ECG. ECG recording in general anesthesia is quite common if acute events are studied, such as potential arrhythmogenicity of drugs or drug candidate substances (Marks et al., 2012; Sakaguchi et al., 2009). ECG recording using needle electrodes can also be useful to monitor cardiac activity during any surgical procedure. The recording of ECG in anesthetized small animals is quite easy to perform. General anesthesia minimizes the stress and ensures minimal movement of the animal (except of the breathing). However, the effect of anesthetics on the cardiac electric activity must be considered (Figure 1B) (Redfors, Shao, and Omerovic, 2014; Sano et al., 2016; Wren-Dail et al., 2017; Liu et al., 2019).

ECG recording in zebrafish represents a specific chapter. In the last two decades, zebrafish became a valuable model in experimental cardiology due to its fast reproduction, low maintenance cost, easy breeding, possibility of genetic manipulation, extensive developmental characterization, and optical transparency (Gut et al., 2017; Duong et al., 2021). Moreover, zebrafish cardiac electric activity is comparable to that of man (Duong et al., 2021). Therefore, several devices for ECG recording in zebrafish were introduced. Most of the approaches require anesthesia or paralysis of the fish. Recently, devices for long-term monitoring of zebrafish were developed and validated (Duong et al., 2021; Le et al., 2022).

If the unwanted effect of general anesthesia may interfere with the experimental purpose, ECG recording in conscious animals is preferred. Even in small animals, the ECG can be recorded using telemetric monitoring (Kramer et al., 2001; Ruppert et al., 2016). Telemetric systems enable to record minimally one lead of ECG in conscious, freely moving animals (Figure 1C). A telemetric unit—microprocessor, battery, and sensors or electrodes for recording of various biosignals—is usually implanted into the abdominal cavity or under the skin. ECG electrodes are fixed subcutaneously. After the implantation, the animal must recover. Then, the ECG can be repeatedly recorded. Such approach is highly valued in long-term studies of drug cardiotoxicity or new drug candidates’ efficiency as well as in chronobiological studies. It usually allows to record the ECG simultaneously with body temperature, arterial blood pressure, or acceleration (movement) of the animal. Such polygraph may uncover subtle dysregulation of the cardiovascular system as well as disruptions of circadian cycles. However, there are some pitfalls which must be considered from the very beginning. Implantation of telemetric unit requires certain surgical skills. Also, the main advantage—recording of the ECG in freely moving animals—is a big challenge. During the movement, skeletal muscles produce a lot of electric potential changes, which cause artifacts in the ECG record. Moreover, telemetric electrodes placed under the skin may slightly change the positions due to body movement. The electrode position change results in the ECG curve change, which may affect ECG analysis. Proper fixation of the electrodes is therefore crucial. Although some approaches promise high-quality ECG recording (Tontodonati, Fasdelli, and Dorigatti, 2011), a standardized, well-reproducible, and broadly accepted procedure of telemetric electrode placement in small animals is missing. In large animals, telemetric monitoring is well established and widely used. Besides implantable telemetric systems, external sensors were introduced. ECG electrodes are placed on the inner surface of a jacket, which is securely fastened to the animal’s trunk. Such fully non-invasive external telemetric system is routinely used in dogs and monkeys (Fish et al., 2017; Skinner et al., 2017). On the same principle, devices for small rodents were introduced. Telemetric ECG recording was used in various wild-life animal species—for instance, in humpback whale (Meijler et al., 1992). Battery lifespan, internal memory capacity, and wireless data transmission speed represent main technical limitations of all telemetric devices. Acquisition price and operating costs may also significantly limit the use of telemetric systems.

If a short-term ECG record is required, non-invasive methods are a good choice. In large well-trainable animals, it is possible to record the ECG by standard stick-on electrodes. Such procedure requires specific training, during which the animal learns to remain motionless for several minutes. In small animals, non-invasive devices usually consist of a platform equipped with electrodes, on which an animal puts its paws (Mongue-Din et al., 2007). In such approach, some restraint of the movement is indispensable. Plastic tunnel restrainers are usually used. But any restraint of movement is stressful for animal, especially for small rodents. To prevent excessive stress, proper handling of the animals must precede the recording. In well-handled rodents, restraint by hand represents a better alternative. Also, mild sedation may decrease animals’ motor activity and therefore increase the quality of the ECG record. Nevertheless, restrained ECG recording technique is a valuable method for rapid screening of several animals in a short time and at low expense. Such method is particularly useful in cardiovascular phenotyping of knockout rats and mice allowing for easy detection of gross abnormalities in cardiac rhythm in a large number of animals (Mongue-Din et al., 2007).

In experimental animals, interpretation of the ECG is usually more challenging than the recording. Lack of standardization in animal ECG recording and less evidence of normal (physiological) values of ECG parameters make the interpretation difficult. The differences in cardiac electric activity among various species also must be considered—especially differences in body size, heart anatomy, and cardiac ionic channel types and their distribution and regulation. The most striking difference in ECG among the species is the RR interval duration (Figure 1A). In mammals, the duration of resting RR interval is directly proportional to the animal’s body mass. The lower the body mass, the higher the resting heart rate. The key cardiac electrophysiological characteristics of various species are summarized in Table 1.

**TABLE 1:** Key electrophysiological characteristics in human and selected experimental animal species. AP, action potential; bpm, beats per minute; ms, milliseconds. *As in ectotherms, the heart rate and duration of ventricular AP in zebrafish vary with body temperature, and numbers indicate heart rate/ventricular AP duration at 28°C and at 19°C (in parentheses), respectively.

	Human	Dog	Rabbit	Guinea pig	Rat	Mouse	Zebrafish
Mean resting heart rate (bpm)	75	70	200	230	300	500	55 (145)*^a^
Ventricular AP (ms)	250	250	120–140	140	50	25–40	143 (311)*^a^
ST segment in ECG	Yes	Yes	Yes	Yes	No	No	Yes^a^

a (Vornanen and Hassinen, 2016) Unless otherwise indicated, adopted from (Farraj, Hazari, and Cascio, 2011).

The ECG of the most frequently used laboratory animals—rat and mouse—is quite distinct from that of humans. There is no distinct ST segment in rat and mouse ECG curves due to short ventricular action potential with minimal plateau phase. The T wave begins immediately after QRS complex. Also, the resting heart rate is significantly higher than that of humans (approx. 500 bpm in mouse, 300 bpm in rat, and 75 bpm in man, respectively). Moreover, significant differences between various strains of the same species were repeatedly reported (Wehrens, Kirchhoff, and Doevendans, 2000; Azar, Sharp, and Lawson, 2011; Konopelski and Ufnal, 2016).

## 3 ECG Analysis

**FIGURE 2:**
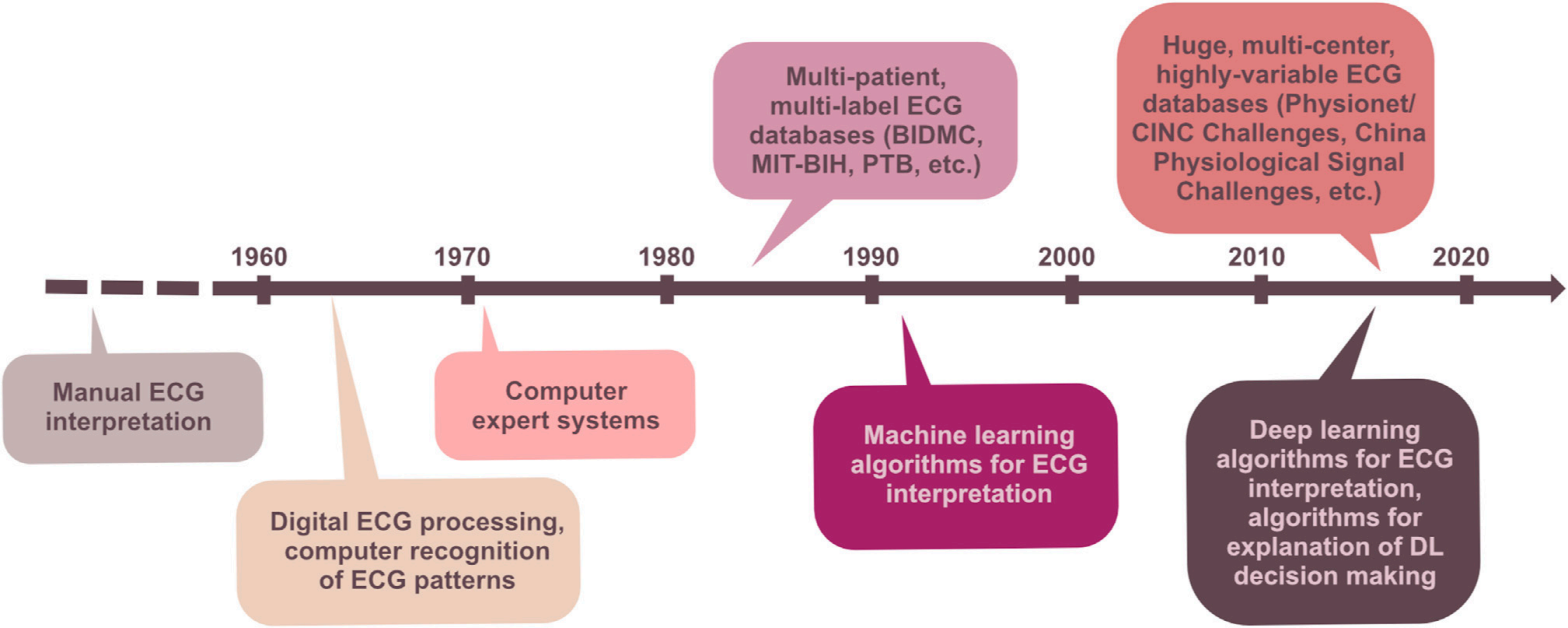
Milestones of ECG processing and analysis in time perspective.

After the ECG is recorded, its pre-processing and analysis may start. The ECG analysis is meant to be a process which gives valuable information about the examined subject (isolated heart model, whole-body animal model, or patient) in terms of cardiac electrophysiology. The early analyses of ECG in the beginning of the 20th century were performed only for measuring time relations in the signal. The introduction of computer technology in the middle of the 20th century opened new possibilities for the analysis of ECG. Despite all the advantages of computer technology, manual evaluation of the ECG by a cardiologist remains an indispensable part of diagnosis. The following paragraphs briefly summarize the development of ECG analysis, the basic principles of the main techniques in clinical and experimental perspectives, and, finally, the main current issues and future directions in this field. Figure 2 illustrates the progress of ECG processing and analysis techniques in time perspective.

### 3.1 Pre-Computer Era

Many physiologists at the end of the 19th century were convinced that the mechanical contraction of the heart is preceded by an electrical action that could be measured. In the first attempts to record electrical potentials of the heart muscle, only heart depolarization and repolarization were recognizable (Waller, 1887). This led to the opinion that such recording would never be useful in clinical practice (Barold, 2003). As signal acquisition techniques have evolved, the quality of the signal has improved, and by the beginning of the 20th century, it was possible to distinguish in ECG five basic deflections: P, Q, R, S, and T waves (Ruiz et al., 2008). The first step in the development of ECG analysis itself was the establishment of a normal recording. At that moment, the first databases of ECG records have been introduced and used to manually measure the durations and magnitudes of the waves (Larsen and Skúlason, 1941; Simonson et al., 1949; Packard et al., 1954).

Along with the improvement of electrical activity recording, the first attempt to quantitatively describe how the heart function is transcribed into the ECG has been done. Numerous equations have been developed to identify the relationships between the heart function and ECG patterns. These equations helped to discover phenomena, which were not visible at the first glance. One of such measures, outlasting in the clinical and experimental use to present, is the QT interval corrected by Bazett’s formula (Bazett, 1920). It reflects the association between the QT interval duration and the heart rate or, in other words, between electrical and mechanical systole of the heart. However, the formula has never been generally applicable; therefore, many other revisions have been developed over the years (e.g., Phang and White, 1943; Krasnoff, 1950).

From today’s perspective, these pioneers have come a long way. The foundations laid by them made it possible to develop the robust and reliable computational methods in the second half of the 20th century.

### 3.2 Computer-Aided Analysis

#### 3.2.1 What Are the Benefits?

Rapid development of computer technologies in the second half of the 20th century led to the extensive automation of various processes traditionally performed by human experts. In medicine, along with analysis of complex imaging data (ultrasound, CT, MRI, etc.), the diagnostics of cardiac disorders intensely uses advanced, highly intelligent computer-aided systems. Probably, the most evident benefit of the computer algorithms is in their ability to process a huge amount of data in a very short time (e.g., Brailer et al., 1997), a process desirable in both clinical and experimental applications. Another advantage is an accurate detection of specific ECG patterns, even in case of mild manifestation or several different manifestations presented in the ECG simultaneously. In such case, the low-resolution, visual diagnostics may be inaccurate or totally false, especially when provided by an inexperienced physician (e.g., Holmvang et al., 1998; Salerno et al., 2003). This limitation, however, may be partially eliminated by using the so-called collective intelligence decision-making system integrated in a special mobile application. Such system enables fast sharing of ECG data and performing the visual interpretation simultaneously by several experts (Hwang et al., 2021). The computational algorithms can analyze multi-lead data simultaneously, for the full length of the record, in a reasonable time, which is not possible by visual inspection. The decision-making process of the computer systems is reproducible, and it is not affected by human factors, such as tiredness and stress (e.g., Taddei et al., 1992). Finally, computerized ECG analysis utilizing widely available telecommunication infrastructure enables using all above benefits in areas with a lack of human experts or in telemedicine applications. In the latter, computer-aided systems play a crucial role by supporting the fast assessment of a huge amount of ECG records (Saeed and Ameen, 2021).

From medical point of view, use of the most advanced methods for ECG analysis offers a simple, widely available, cheap diagnostics tool. By accurate, sensitive, fast, and robust detection of pathological patterns in the ECG, these methods contribute to early diagnosis, selecting the correct treatment strategy, better outcomes, and improved life quality of the patients on the one side and to minimized mental and time demands of clinical staff and decreased national healthcare financial resources on the other side. Automatic prediction systems are also useful in preventive medicine.

Of course, computer algorithms make errors, especially when a low-quality signal or signal with unknown (at least by the algorithm itself) abnormality is analyzed. The computer more likely fails when similar manifestations correspond to different pathologies. In contrast to the computer algorithms, human experts use clinical information about the patient and intuition. Therefore, all statements of the computer systems must be over-read by skilled physicians (Smulyan, 2019). Nevertheless, the decision support system based on the highly accurate algorithm may significantly improve the accuracy of physicians in ECG interpretation, as has been shown previously on myocardial ischemia detection (Tsai et al., 2003).

#### 3.2.2 Basic Pipeline: From Digital Filters to Deep Learning Models

**FIGURE 3:**
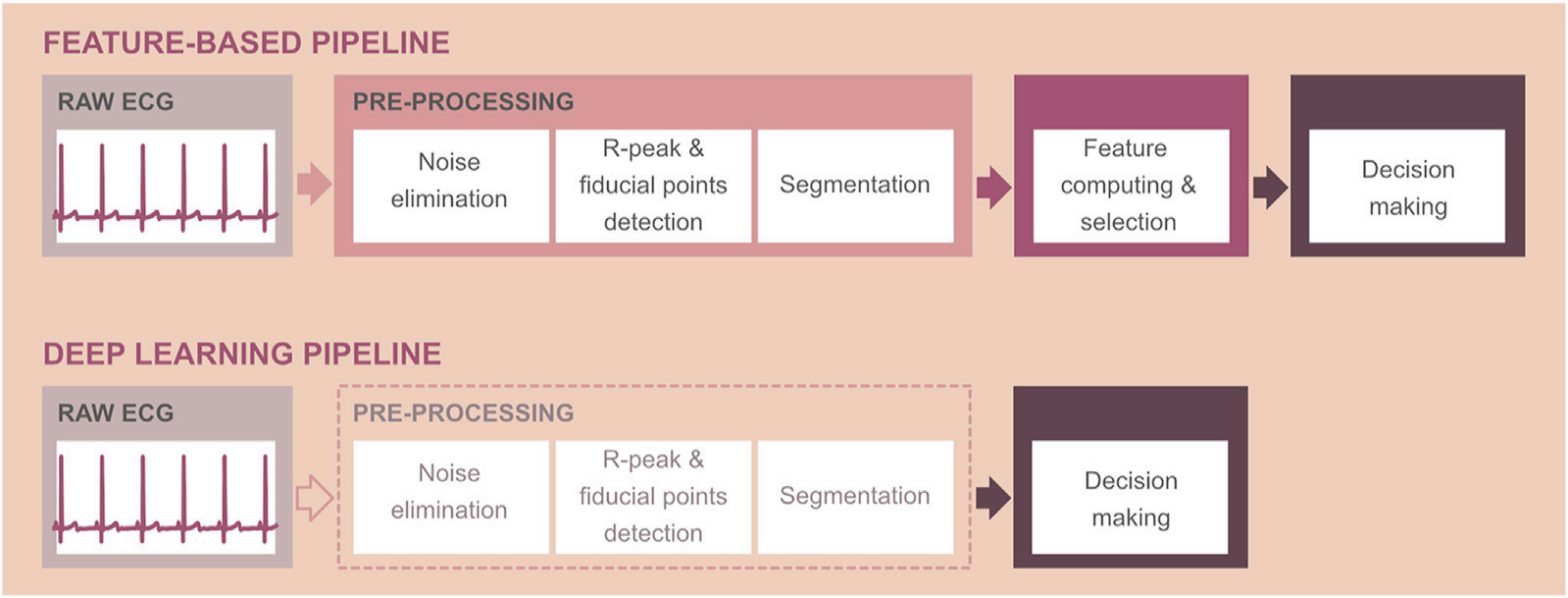
Schematic representation of computer-aided ECG interpretation using feature-based technique (top) and deep learning approaches, which do not require the calculation of ECG features (bottom). Note: pre-processing steps are optional when using deep learning methods and depend on the application.

Traditional computer-aided ECG interpretation consists of the following steps: pre-processing, computation of so-called features, selection of the most relevant ones, and, finally, decision-making (Figure 3, top). ECG pre-processing usually includes suppression of noise, detection of QRS complexes and/or other fiducial points important for ECG delineation, and segmentation (e.g., Berkaya et al., 2018; Kumar and Komaragirikumar, 2018; Vidhya and Jerritta, 2022). Noise of physiological, environmental, or technical origin complicates the interpretation of the records and, thus, must be removed by appropriate methods. Most computer-aided systems use digital filters to solve this problem (see below). Noise-free ECG is suitable for accurate detection of the waves, which usually starts by detection of the most prominent deflection in ECG-QRS complex and continues by searching the peaks and onsets/offsets of the other waves in areas surrounding QRS. In some applications, the outputs of the delineation process are then used for signal segmentation into the separate beats, intervals, or waves of interest. In the next step, the most important in the whole pipeline, the quantitative descriptors—features—are calculated from the initial filtered ECG or the segments. For effective and accurate ECG interpretation, only the most relevant and informative features should be considered. Therefore, the techniques, providing careful selection of the most discriminative features or removing the irrelevant and redundant ones, are applied prior to decision-making. The final step—decision-making—is a process, which must solve specific problems. The most frequent issues related to ECG analysis are the prediction or identification of the cardiac abnormality or localization of cardiac pathological events. Nowadays, these problems are addressed by four different ways: 1) by visual or simple statistical-based expert inspection of the features using established criteria (i.e., by comparing the feature value with the physiological range); 2) by using rather simple, but transparent, human-like expert systems providing automatic analysis of the features *via* decision rules pre-determined in cooperation with clinician experts; 3) by using machine learning (ML) tools—so-called supervised learning algorithms, which are able to learn the interpretations from training data equipped with ground truth labels with no need for expert rules; and 4) by using state-of-the-art tools—so-called deep learning (DL) models.

Feature-based decision-making is usually provided by ML models, from simple linear discriminant analysis (LDA) or logistic regression to more advanced k-nearest neighbor (k-NN), decision tree, random forest, support vector machine (SVM), multiple-layer perceptron (MLP), etc. From mathematical background, most are based on using one or combination of several linear or non-linear equations, which map current input data (ECG samples or features) into the predicted output (label/diagnosis) (for further information, refer to Duda et al., 2000; Chazal et al., 2004; Osowski et al., 2004; Ince et al., 2009; Mishra and Raghav, 2010; Theodoris and Koutroumbas, 2009, etc.). In fact, they solve the classification problem by assigning each input sample (i.e., patient’s ECG) to some of the pre-defined categories (e.g., normal and pathological). ML methods try to find the decision boundaries for identification of different groups of data by learning on samples with known expert interpretations. During the training process, the equation coefficients (model parameters) are adjusted by some special optimization algorithm in order to ensure mapping as accurate as possible (in other words, to ensure correct predicted output of the model). A model with optimal parameters is then able to accurately interpret new, previously unseen, data.

**FIGURE 4:**
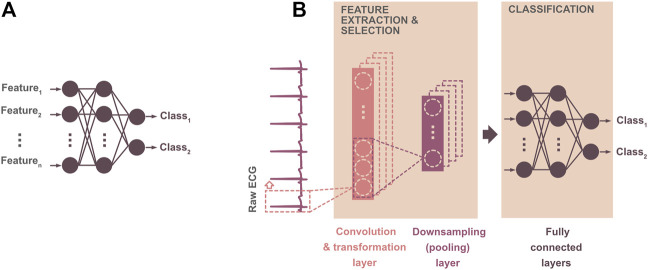
Illustration of two binary classification models: **(A)** straightforward artificial neural network—machine learning model providing ECG classification based on the previously derived features; **(B)** 1D convolutional neural network (1D CNN)—deep learning model providing automatic extraction of the features from raw ECG by convolutional layer(s) and further assignment of the input into the class by fully connected perceptron-like layers. In a 1D CNN model, the convolutional layer consists of filters, which derive important features from the input ECG. The convolution output—feature map—is usually transformed using a linear or non-linear function (in order to simplify the training process and avoid the problem of vanishing gradient) and downsampled by calculating the average (average pooling) or by selecting maximal (max-pooling) values from the feature map. The pooling procedure leads to the reduced number of model parameters and, thus, decreased computation demand.

Both ML and DL methods are parts of artificial intelligence—a wide branch of computer science focused on developing smart systems capable of mimicking human reasoning and solving the tasks traditionally requiring human intelligence. Deep learning is the most recent, advanced technique, extracting highly relevant complex patterns from raw ECG by itself, with no need for feature extraction (see Figure 3, bottom). Briefly, DL neural network models include specific layers for automatic extraction of feature maps from the input signal and layers providing the output prediction (see Figure 4B). Thus, DL models can be considered “all-in-one” solutions, analyzing data in a broad context and outperforming most of existing approaches in terms of time and computational requirements, as well as achieved results (e.g., Goodfellow et al., 2016; Kashou et al., 2021; Ravì et al., 2017). Reduced computational demand of the DL decision-making is caused by both the absence of pre-processing steps and fast interpretation of new data once the model is trained. Compared to the shallow MLP, the DL neural network consists of many neuron layers with different specialization (compare Figures 4A,B). Both shallow and deep neural networks are based on the neurons—nodes—connected to each other in such a way that the input information goes through the deep structure to the last layer, where the predicted output is generated. Each neuron is represented by a linear or non-linear equation, which maps current input data into the output. The neuron parameters—so-called weights—are actually the coefficients of the neuron equation, which are set during the optimization procedure (i.e., during model training). The mathematical operations involved in this process are rather simple: convolution between ECG samples and filter coefficients when extracting the features, linear or non-linear transformation of convolution output, linear combination between current input and neuron weights when generating predicted output, etc. (see Figure 4B). Nevertheless, the use of many highly coordinated neuron layers allows achieving state-of-the-art results.

Two model architectures—convolutional neural net (CNN) with feedforward information flow and recurrent neural net (RNN) with some feedback connections—are mostly used. CNNs process the input with a set of special filters to extract hidden, high-resolution patterns (Murat et al., 2020; Xue and Yu, 2021). RNNs capture the temporal relationships within the entire time-series data (Murat et al., 2020; Xue and Yu, 2021). Since both techniques have some pros and cons, many modifications have been recently introduced. For example, the long short-term memory (LSTM) network was developed to deal with the vanishing gradient problem, which leads to insufficient updation of the network weights as the training process goes through deep layers, as it is characteristic for traditional RNNs (Murat et al., 2020; Xue and Yu, 2021). More recently, both models are used together as a hybrid CNN–LSTM solution (see below) learning from the long-term, complex representations of heart activity patterns created by convolutional layers. DL models in clinical and experimental applications significantly improve the diagnostic yield of routinely used ECG. To create a well-functioning robust DL model, however, large, relevant, and highly variable ECG datasets are needed, as will be addressed below.

#### 3.2.3 ECG Pre-Processing

##### 3.2.3.1 Clinical Perspective

The clinical ECGs are usually corrupted with various types of noise, which may lead to errors in visual inspection and to inaccurate function of automatic delineation and interpretation algorithms. The low-frequency baseline wander, power-line interference (50/60 Hz), and high-frequency noise are those seen in raw ECG. The first one is primarily caused by respiratory movements or poor skin–electrode contact and may cause inaccurate measurement of ST segment, wave amplitudes, R-peak detection, etc. The power-line interference of high magnitude makes the analysis of low-voltage P waves impossible. The high-frequency noise, representing the myopotentials generated during skeletal muscle contraction, complicates the ECG delineation. Digital filters, implemented in a high-pass, band-pass, or low-pass mode with appropriate cut-off frequencies, are usually used to suppress above disturbances in the ECG (Satija et al., 2018; Vidhya and Jerritta, 2022). The desired characteristic of the filter is a zero-phase shift to avoid the distortion of the signal. Special attention should be paid to elimination of the noise with frequency overlapping with the ECG spectrum. For example, inappropriate filtration of power-line interference may lead to undesired alteration (decreased amplitude or even altered morphology) of P waves and QRS complexes. Another way to eliminate noise is the ECG decomposition by so-called wavelet filters (Park et al., 1998). Besides, ML- and DL-based filtering techniques, enabling extra improvement of filtered signal characteristics, have been recently introduced (Xue and Yu, 2021).

After proper noise elimination, the R peaks and/or other fiducial points can be detected. Probably, the most famous and frequently used is the rather robust and simple Pan–Tompkins algorithm (Pan and Tompkins, 1985). Many other R-peak detectors, using searching in the initial ECG or ECG previously transformed to highlight the QRS complexes, have been developed (e.g., Liu et al., 2015; Biran and Jeremic, 2020; Walia and Kaul, 2020). However, none of them is error-free; false detections lead to false alarms and thus increase the demand on intensive care units’ staff (Plesinger et al., 2016). Recently, it was shown that the false R-peak detections can be eliminated by additional validation using the advanced CNN algorithm (Silva et al., 2020).

Complete delineation of ECG can be performed by the CNN–LSTM model with a sensitivity of 97.95% (Peimankar and Puthusserypady, 2021). Another study reports use of sample-wise, so-called semantic segmentation of the raw ECG via the CNN–biLSTM network, with an overall accuracy of 95.54% (Londhe and Atulkar, 2021). Bidirectional LSTM (biLSTM) layers are the layers, where the input sequence is analyzed in the forward and backward directions resulting in better learning of important patterns. Sample-wise technique seems to have great potential in real-time applications due to its robustness and effective dealing with the continuous signal.

Another interesting study was focused on creating the mathematical models able to “extract” the waves from the ECG to make further detection of the fiducial points easier (Rueda et al., 2021). This method outperforms other recent approaches in delineating P and T waves, which are well-known as hardly recognizable and analyzable. The advantage of this algorithm against ML/DL solutions is in its robustness (in terms of required amount of training data and abnormalities present in data) and transparency (in terms of physical meaning of the process used to generate the model and delineation output itself).

Fatimah et al. (2021) compared two segmentation methods: 1) based on R-peak detection and selection of a predefined number of samples before and after the peak and 2) based on the splitting of raw signal into the frames of predefined length. Both approaches are reliable, but the second one is more suitable for real-time applications due to low computational demand.

##### 3.2.3.2 Animal Perspective

Almost all existing methods for ECG pre-processing and analysis have been initially established for human signals. The morphology characteristics of animal ECG are like human ones. However, some differences in heart rate and ECG wave amplitudes and duration exist due to differences in ion channel expression (Bartos et al., 2015) as well as anatomical configuration and innervation of the heart. These differences should be considered when adapting the processing methods to animal applications. Little information is available regarding the processing methods, primarily due to the use of commercial, user-friendly, pre-set software. Only a few research groups develop their own algorithms for ECG pre-processing. Below, the most widespread techniques are presented.

The median filter can be successfully applied on clinical and experimental ECG data to remove low-frequency baseline wander, which complicates the signal delineation and decreases the reliability of morphology features (Steenkiste et al., 2020). For elimination of broad-band noise (such as muscle activity) in the ECG, the above-mentioned decomposition of the entire signal by discrete wavelet transform and reconstruction of “clean” ECG after removing some components containing noise is more suitable than standard linear filtration (Zhang et al., 2019). This method, however, can be sufficient in case of any type of noise, such as a high-frequency one, as shown by Steenkiste et al. (2020) in equine data application.

The narrow-band signals created by ECG wavelet decomposition are often used for accurate detection of waves and QRS complexes. First, signals with well prominent waves of interest are selected, and then the complexes are detected by threshold, which can be constant or adaptively changing according to current signal maximum amplitude or variability. This QRS detection technique was shown as more effective and accurate (sensitivity of 98% and positive predictive value of 99.1%) than the Pan–Tompkins algorithm (sensitivity of 91.5% and positive predictive value of 81.5%) in equine ECGs (Steenkiste et al., 2020). This technique can be used for complete delineation of the entire ECG (with an overall sensitivity of 99.87% and positive predictive value of 99.89%), as presented by Hejč et al. (2015) on data from isolated rabbit heart. Similar detection techniques were recently applied on the ECG recorded in larval zebrafish (Barret et al., 2021). They had to be adapted periodically since significant motion artifacts were present in data and heart rate was changed during the experimental protocol.

Kozumplík et al. (2014) introduced the QRS detector based on the estimation of signal envelope by the Teager–Kaiser energy operator and tested it on isolated rabbit heart data with a sensitivity of 99.84% and positive predictive value of 99.98%.

The first derivative of ECG and further searching for rapid deviations can be used for complete delineation of the signal as shown in detail by Huang et al. (2021) in equine ECG. Simple delineation algorithms are accurate in noise-free data. Otherwise, more robust adaptive techniques must be used, or the signal is segmented, and noisy parts are removed prior to further analysis. This can be solved by algorithms estimating ECG quality. Promising real-time algorithms offer studies by Smital et al. (2020) and Nardelli et al. (2020). These approaches can distinguish several levels of signal quality as evaluated on both human and horse ECGs.

#### 3.2.4 Feature-Based Techniques

Features should represent well ECG patterns characteristic for investigated phenomenon (normal heart function, arrhythmias, drug-induced or stress-induced changes, structural alterations of the myocardium, etc.). The quantitative representation of heart electrical activity by a limited number of highly specific features allows to construct the efficient, robust, fast, and reliable methods for automatic identification and differentiation of various cardiac conditions, as will be shown below. Generally, three main groups of “hand-crafted” ECG features are used in computer-aided systems (e.g., Berkaya et al., 2018; Saini and Gupta, 2021; Gupta et al., 2021a):

- Time-domain features primarily representing 1) the rhythm character of the signal in terms of heart rate and duration of some important intervals (i.e., PQ, QT), 2) the morphology of ECG curve in terms of amplitude, duration, area under the curve, direction, and other shape-related characteristics of the particular waves, and 3) the statistical characteristics of ECG distribution (mean value, standard deviation, kurtosis, skewness, etc.).
- Frequency-domain or time–frequency domain features creating the new representation of ECG by using special transformation (such as Fourier transform (FT), short-term Fourier transform (STFT), and continuous or discrete wavelet transform (CWT/DWT)) and exploring the ECG spectral content and its changes in time.
- Non-linear features revealing non-linear, complex, dynamic character of ECG (hidden to the above methods) based on the chaos theory and information theory (i.e., Lyapunov exponents, Shannon entropy, correlation dimension).

Significant temporal features, intensely used in various areas, are those describing the heart rate variability (HRV). Initially, HRV analysis was introduced to investigate the vago-sympathetic balance and corresponding phenomena (Task Force of the European Society of Cardiology and the North American Society of Pacing and Electrophysiology, 1996, later Task Force Society, 1996). However, the HRV features have been also shown as useful for detection of cardiac arrhythmias, as—technically speaking—they measure the irregularity of RR intervals (or heart rate) (e.g., Task Force Society, 1996; Bernston et al., 1997; Parsi et al., 2021a). These features are calculated from the RR interval sequence (tachogram) and can be divided into three categories, i.e., time-domain (various statistical measures, such as mean or median value, standard deviation of normal interval (SDNN), root mean square of successive RR differences (RMSSD), and triangular interpolation index calculated from the histogram (TINN)), feature-domain (power and peak of very-low-, low-, and high-frequency bands, their ratios, etc.), and non-linear (parameters calculated from the Poincaré map, approximate or sample entropy, detrended fluctuation parameters, complex correlation measure, etc.) ones (Bernston et al., 1997; Tarvainen et al., 2014).

The features should reflect the studied issue. The most relevant, informative, and reliable features can be selected based on the empirical knowledge and experience of the experts, as is common in clinical routine. In computer-aided systems, special computational tools help to identify and eliminate the redundant features or those with poor ability to represent the patterns of interest. The latter often explore the inter-feature relationships (correlation analysis, principal component analysis (PCA), etc.), discriminating abilities of separate features (two-sample tests of statistical difference, as a *t*-test and others), or their combinations (linear discriminant analysis (LDA), decision tree, etc.) or searches among the feature set until the combination leading to the best performance of the model is found (forward or backward selection, sequential floating forward selection, etc.) (Guyon and Elisseeff, 2003; May et al., 2011). The reduction of feature number is desirable: it decreases computational cost and time cost of the method, enables using a simpler model, avoids the problem of model overfitting, improves model generalization, and often increases transparency and interpretability of the whole decision-making process (Duda et al., 2000; Guyon and Elisseeff, 2003, May et al., 2011; Theodoridis and Koutroumbas, 2009).

Feature-based diagnostics tools have been used for several decades and still play a crucial role in clinical and experimental research, primarily due to their transparency. Below, recent ECG applications intensely using these methods are reported.

##### 3.2.4.1 Clinical Perspective

Fatimah et al. (2021) introduced detector of myocardial infarction using entropy, kurtosis, and energy, calculated from one-channel ECG decomposed by FT and ML classifiers, where k-NN achieved the highest accuracy (of 99.96%) outperforming other existing one-channel approaches.

Multi-lead ECG can be used to accurately detect left ventricular hypertrophy, which is a less expensive, less time-consuming, and widely available alternative to the golden standard imaging techniques. Morphological features (R and S waves or ST segment changes, QRS complex inversion, etc.) extracted from CWT-transformed ECGs perform well with ML classifiers, with the most promising accuracy (up to 97.8%) using multi-layer perceptrons (Jothiramalingam et al., 2021).

One example of using the morphology features derived from P–QRS–T segments is the recognition of different beat types, such as normal, supraventricular, and ventricular ectopic beats and fusion of ventricular and normal beats. The SVM model trained on these features performs with overall accuracy 97.8% (Zhu et al., 2019). The arrhythmia-related features can be calculated as mean, maximal, median values, etc., of ECG components derived by WT (Sangaiah et al., 2020).

A novel tool based on the multi-lead ultra-high-frequency ECG and QRS complex features has been recently introduced. It provides the quantitative description of ventricular dyssynchrony with a great potential for selecting the patients for cardiac resynchronization therapy and improving the therapy application (Jurak et al., 2020). One of the proposed features seems to be more suitable for monitoring of depolarization patterns during the biventricular and His-bundle pacing, than common QRS duration.

A recent prospective study presents temporal, spectral, and non-linear HRV parameters as the valuable prognostic tool for evaluation of mortality risks in patients with coronary artery disease (Vuoti et al., 2021).

The most challenging recent application using HRV features is atrial fibrillation (AFIB) detection (e.g., Smisek et al., 2018; Murat et al., 2021a). Timely prediction of paroxysmal AFIB episodes using seven novel Poincaré map features achieves the accuracy over 86% for different ML models and even higher accuracy (98%) when combining with standard HRV features (Parsi et al., 2021b). McCann et al. (2021) studied ECG records from patients undergoing catheter ablation. They reported the lowest AFIB organization level in patients with failed AFIB termination during ablation using instantaneous frequency and adaptive organization index.

Novel multi-scale entropy has been shown as an accurate HRV-based tool for prediction of the malignant ventricular arrhythmia, even using a simple random forest classifier (Chen et al., 2021). Standard non-linear HRV features combined with those from 2D image representation of ECG beats can separate healthy subjects from patients with ventricular arrhythmia with an accuracy of 99.99% by using an ensemble of different ML models (Mandal et al., 2021).

A method based on only six time- and frequency-domain HRV features and a simple k-NN classifier can predict the sudden cardiac death from five-minute RR interval signals recorded by an implantable cardioverter defibrillator with an accuracy of 91.5% (Parsi et al., 2021a).

The random survival forest (RSF) ML model was used to predict spontaneous ventricular tachycardia and ventricular fibrillation events in young and adult patients with long QT syndrome (LQTS) (Lee et al., 2021). The model was trained using the combined feature set including ECG features, family history of LQTS, and occurrence of other arrhythmias. As compared to the statistical multivariate Cox regression model, the RSF model achieved more accurate results with the best precision of 0.95 and sensitivity of 0.93. In an extended multi-ethnic study, the RSF model has been also shown as a promising complex solution for predicting six different cardiovascular outcomes, including all-cause death, stroke, cardiovascular disease, coronary heart disease, atrial fibrillation, and heart failure (Ambale-Venkatesh et al., 2017). In this case, ECG features were combined with imaging features, serum biomarkers, demographic characteristics, and others in order to achieve accurate results, which outperform the output of standard cardiovascular risk scores.

##### 3.2.4.2 Animal Perspective

The basic temporal ECG features such as heart rate and PR and QT interval duration as well as QRS complex duration are routinely used to monitor the character of impulse conduction through different parts of the heart. The assessment of the combination of these features is especially useful when evaluating the possible effects of drugs.

Detailed analysis of QT duration in rabbit protocols with drug-induced long-QT or short-QT syndrome indicated the possible effect of acute mechano-electrical function on long-QT syndrome–related arrhythmogenesis (Nimani et al., 2021). Generally, this feature is a basic marker used in a wide range of proarrhythmic research studies, including those conducted in transgenic rabbit models (Baczkó et al., 2020), larval zebrafish models (Barrett et al., 2021), and guinea pig isolated heart models (Vesely et al., 2019). So-called QT/RR coupling and—though rarely used—HRV analysis can be successfully evaluated in such studies, as previously shown in the context of haloperidol administration in isolated Sprague Dawley rat and guinea pig models (Janousek et al., 2017; Vesely et al., 2019). The prolonged QT duration was found in the methylazoxymethanol acetate rat model of schizophrenia, which has indicated the models’ applicability for investigating the risk factors of ventricular arrhythmias and sudden cardiac death in patients treated with antipsychotics drugs (Stracina et al., 2016).

HRV in the isolated heart model can serve to investigate the intrinsic mechanisms of the cardiac rhythm regulation with no effect of sympathetic/parasympathetic factor (Janousek et al., 2014). Spectral HRV parameters are intensely used in toxicological studies with rodent models (Rowan et al., 2007). Time- and frequency-domain HRV features were recently used to evaluate response of cardiac autonomic modulation in mice to spontaneous and pharmacologically induced vulnerability to cardiac arrhythmias in the context of age-related changes (Piantoni et al., 2021). Due to this simple analysis, the mouse model has been shown as valuable in translational research of age-related risk of arrhythmias. In a recent study (Omoto et al., 2021), the heart rate fragmentation, representing ultra-rapid patterns of HRV, was newly shown as a reliable marker for evaluation of myocardial infarction impact on the cardiac hemodynamic parameters in a Wistar rat.

According to comprehensive overview by Mitchell and Raghav (2021), HRV analysis is commonly used to diagnose cardiac arrhythmia in a horse (in rest, during exercise, or during the treatment). Alexeenko et al. (2020) used two string measures representing the heart rate complexity to predict the paroxysmal atrial fibrillation in equine athletes.

The TINN feature has been found as associated with severity of myxomatous mitral valve disease in dogs (Rasmussen et al., 2012) and was analyzed to evaluate a risk of ventricular tachyarrhythmia and sudden cardiac death (Spier and Meurs, 2004). The Poincaré plot helped to investigate the non-linear geometrical patterns characteristic of dogs as compared to humans and to describe the specifics of impulse conduction through the sinoatrial node (Moise et al., 2020).

Morphological features were found to accurately detect ischemia in the Langendorff-perfused isolated rabbit heart with normal anatomy as well as spontaneously increased left ventricular mass (Ronzhina et al., 2017). The rhythm- and morphology-wise features were used to assess the effects of widely used voltage-sensitive dye di-4-ANEPPS on heart electrical activity in the same experimental model (Ronzhina et al., 2021). Particularly, analysis of the rhythm features revealed the modulation effect of the dye on impulse conduction through the atria, AV node, and ventricles.

Novotna et al. (2017) investigated the usefulness of simply derived and robust features based on high-frequency components of QRS complexes (the maximal peak of QRS envelope, distance from QRS envelope maxima to R peak, and root mean square of the QRS area) to evaluate the conduction velocity in the working isolated rabbit heart under induced hemodynamic changes.

The use of ML-based methods in experimental or veterinary context is usually limited by a small amount of collected data. In case of long-term experiments, however, these techniques could be very helpful to carry out fast, robust, and reliable analysis. For example, Marsanova et al. (2017) differentiated normal sinus, ischemic, and ventricular premature beats recorded in the rabbit isolated heart during experiments with induced global ischemia by morphological and spectral ECG features and various ML models with the best accuracy of 98.6% for k-NN.

Huang et al. (2021) introduced the detector of paroxysmal atrial fibrillation in horses, which uses three consecutive intervals, QT, TQ, and RR (derived from rest, one-channel ECG), and k-NN classification algorithm.

#### 3.2.5 All-In-One Diagnostics Solutions Based on Deep Learning

Many “all-in-one” solutions are working with a raw ECG or the pre-processed, noise-free ECG or 2D “image” representations of ECG created by converting the 1D signal into the so-called spectrogram (via STFT, CWT, etc.) or, more straightforwardly, by saving the ECG segments in some image format for further analysis. These solutions often use the hybrid model, when the first part (e.g., CNN, autoencoder) extracts the most reliable features from the input and the second part (e.g., LSTM net, fully connected layers) provides the final decision-making (classification, prediction, etc.).

Here, the overview of the most recent DL approaches is introduced. To our best knowledge, there is no all-in-one DL solution in experimental physiology applications due to limited data sources. We believe that this gap will be removed soon due to transfer of learning techniques we address in the last section and illustrate the example from the veterinary field.

##### 3.2.5.1 Clinical Perspective – Focused Applications

Many approaches are focused on specific problems, which allows to reach promising results with a relatively simple model architecture and low time and computational requirements even in case of limited data amount. Great improvements based on DL method use were achieved in the detection of acute myocardial infarction and stable ischemic heart disease, with the detection accuracy in the range of 83–99.9% for different model configurations (CNN, ResNet, CNN–biLSTM) (Hinai et al., 2021). Myocardial infarction localization can be recognized with an accuracy of 90.20%, 99.67%, and 99.87% for biLSTM, 1D-CNN, and DenseNet, respectively (Tripathy et al., 2019; Xiong et al., 2021). The ResNet model is a CNN with residual blocks, which allows the signals to pass through several layers in the network resulting in improved training capability of the model with no information loss. DenseNet—network with so-called dense connections—“reuses” the information from each layer by sending it directly as the inputs of all subsequent layers and, thus, maximizes the flow of important inter- and intra-lead patterns between the layers. Adding dense connections increases the total number of trainable parameters in the network and thus increases the computational demand of the method. On the contrary, the overall performance of the network improves. All the above DL approaches outperform existing ML techniques based on morphological features combined with k-NN or SVM in terms of accuracy as well as time required for analysis of new patients’ data (Tripathy et al., 2019; Xiong et al., 2021).

The accuracy of left ventricular hypertrophy detection reaches up to 85.8% when using CNN-extracted ECG features in combination with six-layer-perceptron–extracted demographic features, which outperform routine Sokolow-Lyon criteria and visual inspection (accuracy of 81.8 and 85.5%, respectively) (Kwon et al., 2020). Khurshid et al. (2021) trained ResNet on a 12-lead ECG to predict the LV mass and reached the output significantly correlating with the MRI-based reference values.

Left ventricular systolic dysfunction (LVSD) in critically ill patients can be accurately (71–76%) detected from ECG, which is less time-consuming and less expensive than usually used transthoracic echocardiography. Additional benefit of the DL method is its robustness and efficiency in both patients with and without atrial fibrillation, which is commonly associated with LVSD (Kashou et al., 2021). The latter increases the potential usability of this novel non-invasive, inexpensive, and fast method in acute care screening.

According to recent meta-analysis (Grün et al., 2021), CNN or CNN–LSTM models can predict heart failure from a standard raw 12-lead ECG with sensitivity 83–100% for different architectures and databases.

AFIB detection and differentiation between terminating and non-terminating AFIB episodes were recently performed via time–frequency ECG representation (by chirplet transform) and 2D CNN–biLSTM classifier with an accuracy of 99 and 75.9%, respectively (Radhakrishnan et al., 2021). The authors stated that this method outperforms those based on other representations (by STFT, CWT, and Stockwell transform) and other model architectures (CNN, attention-based, etc.). In another report (Rahul and Sharma, 2022), the AFIB detector using STFT representation and biLSTM model slightly outperformed the method using raw ECG (accuracy of 99.84 and 98.85%, respectively).

According to interesting retrospective research (Attia et al., 2019), an eight-lead ten-second ECG recorded during the sinus rhythm can be successfully used to predict the outcome in AFIB patients *via* the ResNet model with the accuracy of 79.4%. This approach offers fast, inexpensive identification of the patients with a high likelihood of AFIB by timely revealing of the structural changes in atria before the presence of any symptoms.

Detection of abnormal cardiac rhythms (in terms of the width of QRS complex, heart rate, and ECG amplitude), which can respond to electrical shock therapy with further expected restoration of normal sinus rhythm and, consequently, normal cardiac pump function, achieved the best accuracy (91.14%) for the CNN model (Hammad et al., 2021).

##### 3.2.5.2 Clinical Perspective—Multiple-Issue Applications

Recently, there is an effort to design approaches for differentiation of dozens of arrhythmias in ECG by only one complex model. The 1D-CNN proposed by Yildrim et al. (2018) recognizes 17 different cardiac arrhythmias from the one-channel ten-second ECG with an overall accuracy of 91.33%, in the real-time mode suitable for further implementation in mobile/cloud telemedicine applications. Haleem et al. (2021) combined the CNN–biLSTM model segmenting the raw ECGs with the 2D-CNN model, which provides final classification of the beats (previously transformed by STFT) into four categories: normal ECG, arrhythmia (97.9% accuracy), congestive heart failure (100% accuracy), and sudden cardiac death (100% accuracy). 1D ResNet can discriminate among six types of ECGs, as recently shown on a huge, unique database containing over 2 million ECGs recorded from more than 1.6 million patients (Ribeiro et al., 2020); this algorithm outperformed the accuracy of the fourth-year cardiology resident, the third-year emergency resident, and the fifth-year medical student. A similar architecture was previously used to detect abnormalities in one-channel ECG in the frame of PhysioNet Challenge 2017 with the top performance among several solutions (Clifford et al., 2017; Hannun et al., 2019).

Cinar and Tuncer (2021) combined the DL model extracting the features with SVM providing the recognition of sinus rhythm, abnormal arrhythmia, and congestive heart failure with an overall accuracy of 96.77%. The LSTM model trained on raw ECGs achieved 90.67%, and conventional feature-based ML models achieved only about 65–68% on the same dataset. By combination of complex DL and simple ML models, the authors obtained the high-performance tool on the one side and decreased computational time and improved transparency of the process on the other side.

According to Mousavi et al. (2021), the ECG can be successfully represented in the way commonly used in natural language processing, where the distinct waves and QRS complexes are considered the words and the whole ECG a sentence. This representation can be next analyzed by the DL model. The main drawback of this approach (as compared to other DL-based methods above) is the need for R-peak detection, ECG segmentation, and creating valid arrhythmia-related vocabulary required for further integer-encoded representation of ECG. This technique, however, performs better (accuracy of 74–97% depending on the database) than many other existing algorithms.

Compared to the CNN, ResNet, and CNN–biLSTM, the hybrid CNN–transformer model combined with temporal ECG features achieved higher performance in recognizing nine different beat types (Che et al., 2021). The transformer net—one of the most recent DL architectures—has been initially created as a compromise between the CNN (image pattern recognition) and the recurrent neural network (time-series sequence pattern recognition). In this model, the relevant features are driven from input data using the so-called attention mechanism. Approaches, using various transformer modifications, achieve promising results in arrhythmia classification experiments on many different ECG databases (e.g., Che et al., 2021; Hu et al., 2021; Natarajan et al., 2021; Nonaka and Seita, 2021; Meng et al., 2022).

However, adaptation of existing complex architectures, initially proposed for image (CNN, ResNet, etc.) or natural language (LSTM, biLSTM, etc.) analysis, has strong limitations as reported by the systematic study of Nonaka and Seita (2021). Novel ECG-target architectures should be developed to achieve high performance in the future.

### 3.3 Current Issues and Future Directions

#### 3.3.1 Transfer Learning: Sharing the Knowledge Among Areas

Transfer learning is a research method in machine learning, where knowledge from one area is applied to solve the different, but still relevant issue. Transfer learning, when applying by proper way, may solve many different problems. One of them is a problem with heterogeneity of patients’ data. It would be practically useful, if once the model is trained on some data (e.g., from healthy subjects), so that it could be directly applied on data with different diagnostics background (e.g., data from patients with rare arrhythmia). Most probably, however, this will not work well due to highly inconsistent character of patterns from two datasets. Nevertheless, successful results can be obtained when training model on one (freely available and extended) dataset and re-training it later on the other dataset consisting of samples of interest (can be small). In this case, the knowledge learned during the first training and saved in the network weights is used for analysis of the second dataset. During the re-training procedure, the weights from the first layers of the pre-trained model (extracting the relevant features from input data) remain with no changes, and only last layers (providing the classification or prediction itself) are re-trained using new data, which is computationally effective. This transferring of knowledge between the domains seems to be a way for effective usage of the models, well-established in other areas, directly in medical applications, with no extra time and source investments. It could be of special importance in problems, where there are not enough data for efficient training of the model.

DL architectures, initially proposed for image analysis, speech recognition, and other data, can be successfully used for ECG interpretation after appropriate modifications, as has been shown in clinical applications. Salem et al. (2018) recognized cardiac arrhythmias in 2D image representations of ECG using the DenseNet model, which was previously pre-trained on a huge image dataset consisting of the images of animals, objects, etc., with an overall accuracy of 97.23%. Similarly, the VGG image processing network was re-trained to detect LVH in multi-lead ECG data (Soto et al., 2021).

Bizzego et al. (2021) showed that the CNN model for heartbeat identification, initially trained on data from healthy subjects, performs poorly in data from cardiac patients. However, re-training of some layers of this network with a small amount of patients’ data results in significantly improved detection performance. Jang et al. (2021) pre-trained the convolutional autoencoder model on unlabeled one-channel ECG signals from one database and used this model later to classify 12 different rhythms in 12-lead ECG. The authors reported better performance when using the pre-trained model (overall F1-score of about 83.5%) than in case where the model weights were initiated randomly (overall F1-score of about 54.3%). Weimann and Conrad (2021) fine-tuned the weights of CNN, which was initially trained on a large publicly available ECG database, by a small set of data recorded in patients with AFIB and obtained improved performance (by 6.57%) as compared to the non-pre-trained model.

The most exciting and beneficial application of transfer learning was shown in animal data analysis, when the standard use of DL methods is strongly limited by a small amount of available data. A few published reports using DL for animal data analysis are focused mainly on image processing, e.g., detection of canine mammary tumor (Kumar et al., 2020), detection of diffuse degenerative hepatic disease in dogs (Banzato et al., 2018), and classification of the thoracic canine radiographs (Banzato et al., 2021). In animal ECG analysis, the study by Steenkuste et al. (2020) demonstrated application of DL methods by involving transfer learning technique. The CNN model pre-trained on human ECGs was used to recognize four beat types (normal, atrial premature contraction, ventricular premature contraction, and artifact) in equine ECG with an accuracy of 97.1% for the re-trained model, which outperforms the non-pre-trained model.

Aston et al. (2019) fine-tuned several pre-trained image-wise DL models (such as AlexNet, GoogLeNet, ResNet-18, SqueezeNet) *via* a newly proposed symmetric projection attractor calculated from two-lead ECG. They were used to distinguish between wild-type mice and Scn5^+/−^ mutant mice suffering from impaired function of cardiac sodium channel.

From all the above, it seems that DL models can learn not only general but also more specific patterns during transfer learning. Although the DL methods have been recently applied exclusively in veterinary medicine where relatively large amounts of data are available, it seems to be reasonable—due to transfer learning technique—to expect expansion of DL models in experimental cardiology.

#### 3.3.2 Imbalanced Data Problem: Minority Is Out of Game

The lack of data often concerns only certain groups of arrhythmias, such as those with rare incidence in the population or paroxysmal ones, where the ECG manifestation cannot be easily recorded during standard clinical examination. During the training process, the model extracts important information from input samples and uses it for final decision-making. In case of a severely imbalanced train dataset (where the categories are distributed unequally), the model will “focus” on the abnormalities from the majority group(s) and will ignore those from the minority group(s). Most of available ECG databases include much more normal sinus rhythm data than abnormal ones and, thus, are imbalanced. There are some techniques which reduce or eliminate this problem and, thus, ensure effective training of the model.

The simplest way to make the size of particular categories equal is resampling of the training dataset, where the majority class is reduced by random selection of the desired number of samples (undersampling) or the minority class is extended by random repeating of selected samples (oversampling). However, removing of samples from the training set may lead to loss of important information. Simple repeating of minority samples will not make the dataset variable and representative and may not ensure effective model training. Therefore, more sophisticated—so-called augmentation—methods are usually applied.

Augmentation techniques generate new training samples by adding some perturbation in data resulting in improved robustness of the model. First, some manipulations can be applied on initial data, such as random scaling, flipping, shifting, and noising ECG, to achieve accurate detection of multiple arrhythmias (Vicar et al., 2020; Nonaka and Seita, 2021; Do et al., 2022). The same application can profit from using the synthetic samples generated from the training ones using intuitive adaptive synthetic data sampling (ADASYN, Virgeniya and Ramaraj, 2021) or synthetic minority oversampling technique (SMOTE, Ketu and Mishra, 2021). Data samples can be generated artificially by specially trained ML or DL models (such as Gaussian mixture model (GMM), generative adversarial network (GAN), LSTM/biLSTM, CNN), as has been shown for time-series ECG (including dependent multichannel signals) and 2D spectrogram applications (e.g., Lima et al., 2019; Brophy, 2020; Hatamian et al., 2020; Hazra and Byun, 2020). Recently, a unique database of more than 120,000 artificial ECGs, generated by the GAN, has been introduced (Thambawita et al., 2021). This model was trained on more than 7,000 real patients’ ECG records.

From above reports, synthetic ECGs are realistic enough to be used in practice and, consequently, contribute to the expansion of high-performance ML or DL techniques in a wide range of clinical and experimental applications. In the latter area, besides all the above, augmentation approaches, based on data generated by mathematical computational models, seem to have special potential. Models of different complexity—from molecular to organ levels—are available (e.g., Guasch et al., 2013; Henderson et al., 2009; Pagé et al., 1986) for generating the relevant, realistic, clearly interpretable electrophysiological data.

#### 3.3.3 Understandable Means Credible: Open the Black Box

Effective interaction of a human expert (e.g., cardiologist) with the computer-aided system requires trust in the computer’s decision-making. Therefore, an adoption of DL-based diagnostic systems in the routine clinical practice is limited, despite their high performance. As a result, there is a big effort in creating the tools helping to uncover the processes behind the model’s prediction. Such “explaining” methods may help the experts to understand the computer-aided tools and to “safely” use their output to make the final decision. Of course, rather transparent feature-based methods with simple ML models (such as linear discriminant function, decision tree) can be used. These are, however, less successful as DL-based systems (see above). It seems to be more suitable to use the high-performance, though non-transparent, DL algorithms and apply additional techniques to “open” the black box. Many different methods and algorithms have been proposed in the last decade to solve this issue.

Probably, the easiest solution is to visualize the outputs from those layers of deep networks, which generate the features from input data (such as convolutional layers in the CNN model, attention layers in the transformer). The generated feature maps can indicate the parts of the ECG playing the most important role in resulting diagnosis (Meng et al., 2022; Nataraja et al., 2021, etc.). The features maps can be additionally simplified by principal component analysis and used for detailed interpretation of the diagnostics model (Murat et al., 2021b).

Most comprehensive explanation can be obtained by special algorithms, such as Shapley Additive Explanations (SHAP), which assigns the importance weight to each sample by exploring the gradient, calculated when the sample enters the model (Soto et al., 2021). These weights can be then illustrated. A similar method—gradient-weighted class activation mapping (GRAD-CAM)—creates the visualization of the gradient through the entire model (Ho and Ding, 2021). Elul et al. (2021) included the spectro-temporal attention (STA) mechanism, which highlights the most important parts of ECG based on the analysis of temporal and spectral information from selected layers of the network. Other, local interpretable model-agnostic explanation technique (LIME) uses another, very simple, linear model (such as linear regression) to explain the local behavior of the black-box complex model around the sample of interest (Hughes et al., 2021).

#### 3.3.4 Huge Amount of Data: Make It Easier With Multiple-Instance Learning

A huge amount of long-term ECG data requires a lot of time for detailed data labeling, which is crucial for accurate diagnosis and for potential use when creating the ML- or DL-based computer-aided systems. The labeling process is extremely time-consuming and expensive. Especially when each beat should be labeled separately, the risk of misinterpretations due to intensive cognitive load or poor experience of the physician is high. Therefore, multiple-instance learning (MIL) enabling model training using only global labels with no beat-wise annotations can be very useful. MIL is a type of supervised learning, which works with labeled bags of data (instead of the labeled instances) (Carbonneau et al., 2018). When properly combining with ML or DL, it can be successfully used for localization of the pathological events in ECG and generation of the detailed annotation reports, if needed, as shown in myocardial infarction detection (Sun et al., 2012), abnormal heartbeat localization (Tong et al., 2021), or premature ventricular contraction localization (Novotna et al., 2020). The accuracy of such methods is usually comparable to that of standard DL approaches, but MIL-based detectors are beneficial due to less strict requirements on the training dataset.

#### 3.3.5 Deep Learning Expansion: Need for Control

The number of DL studies focused on cardiac arrhythmia detection and classification has intensively grown during the last five years, most probably due to the PhysioNet/Computing in Cardiology Challenge conducted in 2017, where an extended, highly variable database was introduced to the wide audience (Clifford et al., 2017). Many challenge participants applied the CNN or LSTM model to address the topic and achieved the best performance in binary (AFIB vs. non-AFIB) and multi-class (sinus rhythm, AFIB, atrial flutter, etc.) issues (Murat et al., 2021a). The growing trend in using DL is still present due to international challenges (PhysioNet/Computing in Cardiology 2020 and 2021, China Physiological Signal Challenge 2018–2021) focused on the algorithms for reliable QRS detection, supraventricular and ventricular premature contraction detection, AFIB detection or paroxysmal AFIB localization in ECG, multi-label classification of 24 different arrhythmias, or identification of the ECG leads with the highest discrimination ability. During the challenges, high-quality, freely available, and accurately labeled databases are introduced to the wide audience, which enables further development of high-performance robust algorithms (Perez Alday et al., 2021). The availability of large ECG databases, however, makes the opportunity for people with no basic knowledge in the cardiac arrhythmia domain to be involved in creating the algorithms. In this situation, one cannot expect correct and careful usage of ML or DL methods. The only way to create trustworthy diagnostic systems, which meet the true clinical needs, is close cooperation among a wide range of experts, such as clinicians, engineers, data scientists, and programmers.

Another issue, which needs to be solved soon, is a lack of the guidelines for creating and evaluating the diagnostic ML and DL algorithms. Bond et al. (2021) formulated this aspect in the context of systems for ECG interpretation as a need for best practices for “stress-testing” algorithms. They recommend to test the novel algorithms under different conditions: using both clean and noisy data, data recorded by misplaced or/and interchanged electrodes, data recorded in different hospitals by different devices and from different ethnic groups, etc. This may ensure creating the reliable, accurate, and robust algorithms useful in clinical routine support. Thus, formation of international working groups focused on the relevant guideline formulation can be expected in the near future. Best practices—after some modifications—will be further integrated in experimental applications.

## 4 Final Remarks

The present article reviews current perspectives of ECG recording and analysis. It presents a unique combination of clinical and experimental points of view. This approach may attract a broad range of readers—not only researchers in the area of cardiology but also biomedical engineers, mathematicians, and last but not least clinicians. Looking at the same topic from various angles and summarizing information obtained from various models may help interested readers of numerous specializations understand each other better. As a result, it might help to plan future research activities of multidisciplinary teams, consisting of both researchers and clinicians.

In the 21st century, recording of electrical activity of the heart muscle in the form of electrocardiogram may appear rather obsolete. The truth is that numerous sophisticated methods enable the researchers and clinicians to obtain information about electrical and mechanical activities of the heart and visualize these processes. On the contrary, new ways of ECG recording together with advanced methods of its analysis open new possibilities for its use.
